# Afrobatrachian mitochondrial genomes: genome reorganization, gene rearrangement mechanisms, and evolutionary trends of duplicated and rearranged genes

**DOI:** 10.1186/1471-2164-14-633

**Published:** 2013-09-21

**Authors:** Atsushi Kurabayashi, Masayuki Sumida

**Affiliations:** 1 Institute for Amphibian Biology, Graduate School of Science, Hiroshima University, 739-8526 Hiroshima, Japan

**Keywords:** Mitochondrial genome, Gene rearrangement, Concerted evolution, Substitution rate, Selection, Afrobatrachia, Ranoides

## Abstract

**Background:**

Mitochondrial genomic (mitogenomic) reorganizations are rarely found in closely-related animals, yet drastic reorganizations have been found in the Ranoides frogs. The phylogenetic relationships of the three major ranoid taxa (Natatanura, Microhylidae, and Afrobatrachia) have been problematic, and mitogenomic information for afrobatrachians has not been available. Several molecular models for mitochondrial (mt) gene rearrangements have been proposed, but observational evidence has been insufficient to evaluate them. Furthermore, evolutionary trends in rearranged mt genes have not been well understood. To gain molecular and phylogenetic insights into these issues, we analyzed the mt genomes of four afrobatrachian species (*Breviceps adspersus*, *Hemisus marmoratus*, *Hyperolius marmoratus*, and *Trichobatrachus robustus*) and performed molecular phylogenetic analyses. Furthermore we searched for two evolutionary patterns expected in the rearranged mt genes of ranoids.

**Results:**

Extensively reorganized mt genomes having many duplicated and rearranged genes were found in three of the four afrobatrachians analyzed. In fact, *Breviceps* has the largest known mt genome among vertebrates. Although the kinds of duplicated and rearranged genes differed among these species, a remarkable gene rearrangement pattern of non-tandemly copied genes situated within tandemly-copied regions was commonly found. Furthermore, the existence of concerted evolution was observed between non-neighboring copies of triplicated 12S and 16S ribosomal RNA regions.

**Conclusions:**

Phylogenetic analyses based on mitogenomic data support a close relationship between Afrobatrachia and Microhylidae, with their estimated divergence 100 million years ago consistent with present-day endemism of afrobatrachians on the African continent. The afrobatrachian mt data supported the first tandem and second non-tandem duplication model for mt gene rearrangements and the recombination-based model for concerted evolution of duplicated mt regions. We also showed that specific nucleotide substitution and compositional patterns expected in duplicated and rearranged mt genes did not occur, suggesting no disadvantage in employing these genes for phylogenetic inference.

## Background

Animal mitochondrial (mt) genomes typically consist of a closed circular molecule 16–17 kilo base pairs (kbp) in size, with multiple copies existing in every cell [1]. Most animal mt genomes contain the same 37 genes: 12S and 16S ribosomal RNA genes (*12S* and *16S rrn*s), 22 transfer RNA genes (*trn*s), and 13 protein-coding genes (ATPase subunits 6 and 8: *atp6* and *8*; cytochrome oxidase subunits I, II and III: *co1–3*; cytochrome *b* apoenzyme: *cytb*; and nicotinamide adenine dinucleotide dehydrogenase subunits 1–6 and 4 L: *nd1–6* and *4 L*) [2,3]. Of these 37 genes, 28 are encoded on the heavier guanine-rich DNA strand (H-strand), while nine are encoded on the cytosine-rich light strand (L-strand). Vertebrate mt genomes also contain a long non-coding region (approximately 0.5–9 kb; [4]) called the control region (CR, or the D-loop region), which includes the signals for regulating mtDNA transcription and the replication origin of the H-strand (O_H_) (e.g., [5,6]). A short non-coding replication origin for the L-strand (O_L_) has also been identified in the mt genomes of most vertebrates, excluding birds [2,5,7].

Nucleotide substitution rates within mt genes are widely accepted to be much faster than in the nuclear genome, and the 37 mt genes generally have different substitution rates from one another [8-10]. Because of their high copy numbers and fast and/or multiple nucleotide substitution rates, mitogenomic sequences have been widely used in genetic and evolutionary studies (e.g., [11]). Nearly 70% of molecular phylogenetic studies on animal taxa have used mt gene data [12].

Mitochondrial gene arrangements tend to be conserved within vertebrates, with all 37 genes and the CR organized in relatively the same order in taxa from teleost fishes to eutherian mammals (e.g., [2]). However, rearranged mt genomes have been found in some taxa of all major vertebrate groups (fishes, amphibians, reptile, birds, and mammals (e.g., [2,13])). Because the animal mt genome has no introns and very few intergenic spacers and is assumed to lack recombination (e.g., [1,14]), rearrangements of its genes have usually been interpreted to be the result of tandem duplication caused by replication errors, e.g., the tandem duplication and random loss (TDRL) model [15,16]. However, recent evidence for recombination in the animal mt genome compels the reconsideration of several other hypothesized duplications and gene rearrangements [11,17-22]. Consequently several gene rearrangement modes mediated by recombination have been proposed [4,23-26].

Two alternative concerted evolution models, based on duplication and recombination mechanisms, have also been proposed to explain nearly-identical nucleotide sequences occasionally found between duplicated CRs [27]. However, observational evidence to validate these models is still insufficient because of the rarity of mt genomes having intermediate conditions in the gene rearrangement process (but see [4,13,26,28,29]). Furthermore, different nucleotide substitution trends, such as the relaxation of purifying pressure and accompanying substitution rate acceleration, have been suspected for (nuclear) duplicated genes (e.g., [30,31]). Also a region-specific nucleotide compositional bias possibly affecting the rearranged genes has been reported from vertebrate mt genomes (e.g., [32-34]).

Substitution-rate and nucleotide-compositional heterogeneities among lineages are well known to cause phylogenetic artifacts (e.g., [35-37]). Therefore, many phylogeneticists are particularly interested in knowing how marker genes evolve [38]. Unfortunately, the evolutionary trends of duplicated and rearranged mt genes in animals have not been well investigated because of their low numbers and, especially, because very few examples exist of rearranged and non-rearranged mt genomes within closely-related taxa. Thus, an understanding of the patterns and mechanisms of mitogenomic duplications and rearrangements and the evolutionary trends of the resulting genes could be fostered by analyzing an animal group with (1) differential frequencies of genome rearrangements among lineages and (2) intermediate states of the genomic rearrangement process. Among vertebrates, anurans (especially Ranoides; see below) are good candidates to meet these conditions.

**Figure 1:**
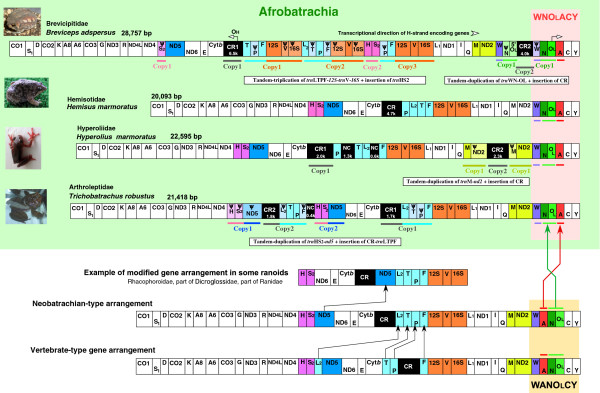
**Mitochondrial genomic organizations of afrobatrachians and other anurans.** The mitochondrial (mt) genomic organizations of four afrobatrachians are illustrated. Vertebrate- and neobatrachian-type mt gene arrangements and an example of the modified arrangement found in ranoids are also shown. Genes, pseudogenes, control regions (CRs), major non–coding regions, and light-strand replication origins are shown in boxes. The H- and L-strand encoded genes are denoted above and below each gene box, respectively. The sizes of the boxes do not reflect the actual lengths of the genes and non–coding regions. Transfer RNA genes (*trn*s) are designated by single-letter amino acid codes. L1, L2, S1, and S2 indicate trns for Leu(UUR), Leu(CUN), Ser(AGY), and Ser(UCN), respectively. “ψ” indicates a pseudogene. The heavy- and light-strand replication origins are abbreviated O_H_ and O_L_, respectively. Other gene abbreviations are: 12S and 16S, 12S and 16S ribosomal RNAs; CO1–3, cytochrome c oxidase subunits 1–3; Cytb, cytochrome b; ND1–6 and 4 L, NADH dehydrogenase subunits 1–6 and 4 L. Colored boxes represent genes, pseudogenes, O_L_, and CR with duplications and/or rearrangements in afrobatrachians. Copies 1–3 show duplicated and/or rearranged genomic regions, and copy 1 indicates the putative original copy. Brief explanations of duplication events are denoted in the open boxes. The transcriptional direction of H-strand encoded genes and the directions of heavy-strand (from O_H_) and light-strand (from O_L_) replications are shown by an open arrow and open arrowhead, respectively. Closed arrows indicate the rearranged genes and the inferred evolutionary directions of the rearrangements.

**Figure 2:**
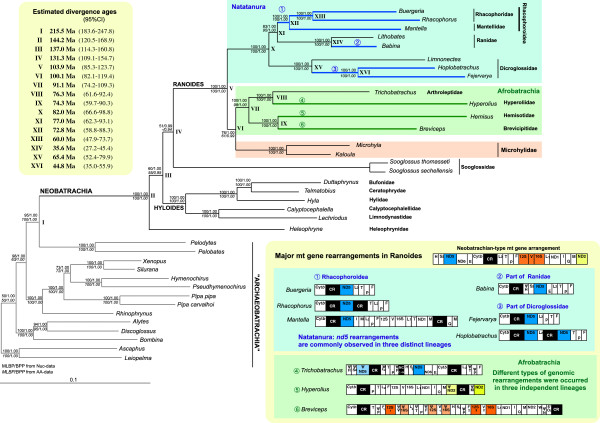
**Phylogenetic relationships of anurans, estimated divergence ages, and major mitochondrial genomic rearrangements in ranoid lineages.** The maximum likelihood (ML) tree of the anurans from the complete mitochondrial (mt) genome and nine nuclear genes (the Nuc–dataset) is shown. Numbers on the nodes indicate ML bootstrap values / Bayesian posterior probabilities for, respectively, the Nuc-dataset and an AA-dataset, in which the coding genes were transcribed. The estimated ages and 95% confidential intervals of 16 nodes (I–XVI) are shown in the upper left box. Circled numbers (1–6) and blue branches on the tree indicate lineages with major mitogenomic rearrangements. Details of the rearrangements in each lineage are shown in the box at lower right.

Generally two major anuran groups, Archaeobatrachia and Neobatrachia, are recognized. The former is regarded as a paraphyletic assemblage of basal anurans (e.g., [39]). The latter is a monophyletic taxon of modern anurans and contains over 95% of extant frogs [40-42]. The mt gene arrangements of almost all archaeobatrachians so far reported (excluding the *Leiopelma archeyi* mt genome [43]) are identical to the typical vertebrate-type arrangement (e.g., [9] and Figure 1]). However, neobatrachians commonly have a slightly modified gene arrangement (neobatrachian-type arrangement) having four *trn*s translocations relative to the vertebrate-type arrangement (e.g., [44,45]), and Figure 1]. In addition, further gene rearrangements have been reported in some lineages of Ranoides, which comprises three major groups: Microhylidae, Natatanura, and Afrobatrachia (see Figure 2). Microhylidae (= narrow-mouth toads) mt genomes have neobatrachian-type arrangements [45,46], but large mitogenomic reorganizations involving duplications and rearrangements of protein-coding genes and CRs have been found from three distinct lineages of Natatanura (= Ranidae, *sensu lato*), including all members of Rhacophoroidea, part of Ranidae, and part of Dicroglossidae ([4,47-49]; and Figure 2]). Mitogenomic information has not been available for the remaining taxon, Afrobatrachia.

Afrobatrachia, endemic to Africa, consists of four families: Arthroleptidae, Hyperoliidae, Hemisotidae, and Brevicipitidae. Historically, the phylogenetic position of Hemisotidae has been problematic, and all breviciptid species were long regarded as members of Microhylidae (e.g., [40,50]). Although recent molecular phylogenetic analyses support afrobatrachian monophyly (e.g., [51,52]), clear synapomorphic characters have not been found for this group [40]. Furthermore, the phylogenetic relationships among the three major ranoid taxa have been somewhat problematic (see Results and Discussion section) and should be verified using sufficient molecular data.

In this study, we analyzed afrobatrachian mt genomes to explore the occurrence of novel mitogenomic reorganizations and to gain new insights into the mechanisms of this process. We also reviewed the phylogenetic relationships of afrobatrachians using the largest molecular dataset yet applied to this group. Finally, we tested several evolutionary trends expected in rearranged mt genomes and in duplicated and rearranged genes using afrobatrachian and other available ranoid mitogenomic information.

## Methods

### Specimens used

To sequence whole mt genomes of afrobatrachians, we used four species representing all four afrobatrachian families: *Breviceps adspersus* (Brevicipitidae), *Hemisus marmoratus* (Hemisotidae), *Hyperolius marmoratus* (Hyperoliidae), and *Trichobatrachus robustus* (Arthroleptidae). Five natatanuran species (*Babina holsti* and *Lithobates catesbeianus*, Ranidae; *Buergeria buergeri*, Rhacophoridae; *Hoplobatrachus tigerinus* and *Limnonectes fujianensis*, Dicroglossidae) were used to analyze their nuclear gene sequences.

Since about 2006, classifications of many frog taxa have been in a rapid state of transition. To avoid needless confusion, in this study we have basically followed the nomenclature and circumscriptions of Frost et al. [40] and Frost [41].

DNA sequencing was performed using previously extracted total DNAs [47-49,53]. Consequently, no animals were used in this study.

### DNA sequencing

Whole mt genomes of the four afrobatrachians were PCR-amplified and sequenced. PCR reactions and primers have been described previously [53]. The primer-walking method was employed for sequencing using an ABI 3130xl automated DNA sequencer (Applied Biosystems, Foster City, CA, USA) with the BigDye Terminator Cycle Sequencing Kit (ABI). PCR fragments containing CR DNA with long tandem repeats and/or mononucleotide tracts that could not be sequenced by primer walking were subcloned into *E. coli* vector pCR-2.1 or pCR-XL using the TOPO TA Cloning Kit (Invitrogen, Carlsbad, CA, USA). To precisely sequence the long tandem repeats, a series of deleted subclones was made from the resultant subclones using the Exonuclease III deletion method [54]. The resulting mt gene sequences were identified by comparison with corresponding gene sequences from other vertebrates. To identify CRs, we looked for conserved sequence blocks 1, 2 and/or 3 (CSB I–III), characteristic elements of vertebrate CRs that are considered to be to be essential for the synthesis of D-loop DNA and for H-strand replication (e.g., [55]). We also found many possible pseudogenes. We identified them based on their > 40 bp lengths and > 50% sequence similarity to their corresponding functional paralogs.

We also amplified and sequenced 1–5 of seven nuclear genes (*bdnf, histone -3a, pomc, rag1, rho, slc8a1,* and *slc8a3*) from each of the four afrobatrachians and five natatanurans. The PCR strategy and primers used for these genes were basically the same as in Irisarri et al. [38], but we made a primer set for *slc8a3* (NCX3_FowN: GARGTCATAACWTCACARGARCG; NCX3_RevN: AAGATATCATCATCRATAATYCC) and a reverse primer for *histon-3a* (H3NR_mod: ATRTCCTTRGGCATRATTGTKAC). The newly determined mt (AB777216– AB777219) and nuclear (AB777220–AB777233) sequences were deposited in the DDBJ/EMBL/NCBI DNA databases.

### Preparation of sequence datasets for evolutionary analyses

To perform phylogenetic and dating analyses, we used our whole-mitogenomic dataset and the sequences of nine nuclear protein-coding genes (*bdnf, cxcr4, histon-3a, pomc, rag1, rag2, rho, slc8a1,* and *slc8a3*). The taxon-sampling strategy basically followed that of the recent comprehensive anuran mitogenomic study by Irisarri et al. [38], but several ranoid taxa with rearranged mt genomes (e.g., *Buergeria*, *Babina*, and *Hoplobatrachus*) were added to analyze the mode of evolution of the duplicated and rearranged mt genes. Because our phylogenetic analyses focused mainly on the family level, and to maximize the completeness of our nuclear gene data, sequences from congeneric species were merged (see Additional file 1) to form a few operational taxonomic units (OTUs) in a similar way to some previous studies (e.g., [38,56-58]). We used 36 and 45 OTUs for phylogenetic and time tree reconstructions, respectively. The 36-OTU dataset comprised only frogs, with *Ascaphus* and *Leiopelma*, which occupy the most basal positions among extant anurans (e.g., [58]), used as outgroups. The 45-OTU dataset also included nine non-frog taxa, i.e., three salamanders, three caecilians, a lizard, a bird, and a mammal, to allow more time calibration points. The details of the taxa and genes used in this study are shown in Additional file 1. Sequence data used in this study are available in Additional file 2.

Mitochondrial and nuclear gene sequences of the 45 OTUs were aligned. For each protein-coding gene, the deduced amino acids were aligned using MAFFT [59] implemented in TranslatorX [60] with the L-INS-i option and default settings. Ambiguously-aligned sites were removed using Gblocks v.0.19b [61] (also implemented in TranslatorX) with default settings. Finally, trimmed protein alignments were used to guide a codon-based alignment of nucleotide sequences. Sequences of mt *rrn*s were aligned using MAFFT with the Q-INS-i option, in which secondary structure information was considered [62]. The mt *trn*s were aligned manually based on their putative secondary structures. Ambiguously-aligned positions in both mt *trn* and *rrn* gene alignments were excluded using Gblocks as described above. The resultant alignments for each gene were used to compare substitution rates and nucleotide compositions among rearranged and non-rearranged genes (*12S* and *16S rrn*s, *nd2*, and *nd5*; see below). The individual nucleotide alignments were concatenated into a single dataset (the Nuc-dataset; 15,233 nucleotide sites in total) and the individual amino acid alignments of the mt and nuclear protein-coding genes were concatenated with the *rrn* (1,921 bp) and *trn* sequences (1,424 bp) into one data matrix (the AA-dataset; 5,944 amino acid and 3,345 nucleotide sites). Previous studies have suggested that long-branch attraction may mislead phylogenetic reconstructions of anuran trees because of the high evolutionary rates of neobatrachian genes (e.g., [63,64]). To minimize such artifacts, third codon positions in the nucleotide dataset were *a priori* excluded from the phylogenetic reconstruction and dating analyses.

The best partitioning schemes for Nuc- and AA-datasets were estimated under the Akaike information criterion (AIC) [65] using PartitionFinder v1.0.1 and PartitionFinderProtein v1.0.1, respectively [66]. For the Nuc-dataset, a seven-partition scheme was optimal: (1) first codon positions of all mt protein genes, (2) second codon positions of all mt proteins, (3) first codon positions of all nuclear protein genes, (4) second codon positions of all nuclear proteins, (5) *12S rrn*, (6) *16S rrn*, and (7) *trn*s. For the AA-dataset, a scheme with 19 partitions was suggested: eight mt protein sequence partitions (5 single mt protein partitions and *atp6/cytb/nd1/nd4L*, *co2/co3*, and *nd2/nd4* partitions), eight nuclear protein partitions (7 single protein partitions and *cxcr4/rag1* partition), plus three mt *rrn* partitions (= partitions 5, 6, and 7 above).

Heterogeneity in nucleotide composition among lineages negatively affects the accuracy of phylogenetic inference (e.g., [37,67]). To avoid this effect, we checked the nucleotide composition homogeneity of all seven Nuc-dataset partitions using Pearson’s chi*-*squared (*χ*^2^) test implemented in phylogears ver. 2–2.0 [68]. Although homogeneity was not rejected (*P* > 0.05) in six of seven partitions, it was rejected for the partition of the mt first codon positions (*P* = 1 × 10^-26^). For this partition, we applied the AC-coding (=RY-coding) method [67,69,70] to eliminate the nucleotide composition bias (*P* = 0.99 after AC-coding). The AC-coded partition was used for phylogenetic tree reconstruction but not in the dating analysis.

Nuc- and AA-datasets and detailed information on gene partitions and substitution models are available in Additional file 3.

### Phylogenetic analyses

Both the Nuc and AA anuran datasets (36 OTUs) were analyzed by maximum likelihood (ML) using RAxML v.7.0.3 [71] and by Bayesian inference (BI) using MrBayes5D [72], a modified version of MrBayes 3.1 [73]. The best substitution models for the nucleotide and amino-acid partitions were estimated using Kakusan4 and Aminosan, respectively [74]. To select the substitution models, we used the AIC for ML analyses and the Bayesian information criterion (BIC) for BI analyses.

The rapid hill-climbing algorithm [75] starting from 100 randomized maximum-parsimony trees was used for ML searches in RAxML, which independently optimized all substitution model parameters in all partitions. For BI, we ran 20 million generations of four simultaneous Markov chains and sampled every 1000 generations. Convergence was checked *a posteriori* using Tracer v.1.5 [76]. The first 10% of generations were discarded as burn-in to prevent sampling before the Markov chains reached stationarity. Support for internal branches was evaluated using bootstrap percentages (BP) from 1000 non-parametric replicates for ML and using posterior probabilities (BPP) for BI.

### Molecular dating analysis

To estimate divergence times of afrobatrachians and other anurans, we used MCMCTree as implemented in PAML 4.6 [77]. This program implements a Bayesian dating method, with a soft-bound approach for age constraints and Cauchy distribution for lower-bound constraints. For this analysis, we used the full Nuc-dataset (46 OTUs) with seven data partitions (see above) and the tree topology from our phylogenetic analysis (Figure 2). The dataset and other setting files used are available in Additional file 4. Independent GTR + Γ models were applied to each of the partitions. We applied seven calibration points suggested from fossil records as priors for divergence time estimations (lower bounds) according to Irissari et al. [38] as follows: 1) > 312 million years ago (Ma) for the Sauropsida-Synapsida split, 2) > 260 Ma for the Archosauromorpha-Lepidosauromorpha split, 3) > 146 Ma for the Cryptobranchidae-Hynobiidae split, 4) > 249 Ma for the Anura-Caudata split, 5) > 161 Ma for branching of Discoglossoidea, 6) > 146 Ma for branching of Pipoidea, and 7) > 53 Ma for the *Calyptocephalella*-*Lechriodus* split. Cauchy distributions were used with default parameters (p = 0.1, c = 1). The Markov chain was run for 11 million generations with sampling every 100 generations, the first 1 million of which were discarded as burn-in. Chain convergence and adequate effective sample sizes (> 200) of all parameters were checked with Tracer [76].

The MCMCTree implemented two different molecular clock models (independent and correlated). To test which model was most suitable for our data, we performed a cross-validation analysis of the standard errors (SEs) of the posterior ages of the seven calibration nodes. Briefly, we ran the program as described above but eliminated one of the seven calibration points; the posterior SE of that node was calculated under both the independent and correlated clock models. The SE calculations were repeated for all calibration points. The sums of the SEs of all calibration points were compared between the two models. The total SE from the correlated clock model (0.00434) was smaller than that of the independent clock model (0.00449), so we adopted the former for our data.

### Relative-rate tests

To compare substitution rates of mt genes among neobatrachian lineages, relative-rate tests (RRTs [78]) were performed using the program RRTree [79]. This program extends the method of Li and Bousquet [80] and compares mean rates between lineages relative to the outgroups while accounting for phylogenetic relationships using topological weighting [81]. Nucleotide genetic distances were estimated with the Kimura two-parameter substitution model [82]. We compared substitution rates of all mt genes, all mt protein-coding genes, and/or rearranged or duplicated genes (*nd2*, *nd5*, *12S*, and *16S rrn*s) among several distinct lineages. Those lineages and the outgroup used for each comparison are shown in Tables of RRTs.

### Detecting changes in selective pressure on mt protein-coding genes in neobatrachians

Several studies of various lineages have shown that non-synonymous/synonymous substitution ratios (dN/dS ratio = ω) can successfully be used to identify changes in selective pressure, including in highly-divergent taxa [38,83,84]). We compared many so-called “branch” models [see [83] with different assumptions about selection coefficient ratios to determine in which frog lineages changes in selective pressure on the mt protein-coding genes had occurred and to understand whether accelerated evolutionary rates (especially for duplicated and rearranged genes) in neobatrachians were due to changes in selective pressure. The codeml program implemented PAML 4.6 [77] was used to estimate the likelihood of the tree (having the Figure 2 topology but including only 22 neobatrachian taxa) and the ω values of the branches under the given models for the dataset of all mt protein-coding genes and for single-gene alignments of *nd2* and *nd5*, which are duplicated or rearranged in some neobatrachian lineages. Branch lengths were first optimized for each dataset assuming a single ω for the whole tree, and they were fixed while the other parameters were estimated. The null model had a single ω value for all branches, while the alternative models allowed unique ω values on one or more designated branches. The alternative models were compared against the null model using the likelihood ratio test (LRT), and all models were compared simultaneously using the AIC [65].

## Results and discussion

### Phylogeny of anurans and divergence ages of afrobatrachians

We first reconstructed a phylogenetic tree for anurans. Figure 2 shows the ML tree (-ln*L* = 134944.28) derived from the Nuc-dataset. The BI tree generated from the same dataset and the ML and BI trees from the AA-dataset recovered the same topology, so we assumed that this was the best phylogenetic hypothesis and used it in subsequent dating and other evolutionary analyses.

Our phylogenetic hypothesis was basically congruent with those of recent phylogenetic studies (e.g., [38,40,51,52]) and confirmed the following relationships for higher anuran taxa. (i) Neobatrachia is monophyletic. (ii) “Archaeobatrachia” is paraphyletic with respect to Neobatrachia. (iii) *Heleophryne* is most basal among neobatrachians. (ix) Ranoides and Hyloides (*sensu* Frost et al. [40]; excluding family Sooglossidae) are both monophyletic within Neobatrachia. (v) The three major clades of Ranoides are Afrobatrachia, Microhylidae, and Natatanura. All of our trees recovered a sister relationship between Ranoides and Sooglossidae (BPPs = 99/94% in Nuc/AA-datasets, respectively), but with low BP supports as in many previous studies [38,51].

Although many alternative hypotheses for the afrobatrachian families exist (see [41]), afrobatrachian monophyly has been suggested by several recent comprehensive studies (e.g., [41,51,52]). Likewise, our results strongly supported afrobatrachian monophyly (BP = 100/98%, BPP = 100/100% in the Nuc/AA-datasets, respectively). We also discovered a synapomorphic mitogenomic structure (a rearranged “WNACY” *trn* cluster shared by all four afrobatrachian families; Figure 1 and see below), although no morphological synapomorphy for afrobatrachians has yet been found [41].

The phylogenetic relationships of the three major ranoid groups (Afrobatrachia, Microhylidae, and Natatanura) have been very problematic. Morphological studies suggested a close affinity of Afrobatrachia and Natatanura [85-87]. In contrast, *rag1* data indicated a sister relationship between Natatanura and Microhylidae [50]. Although recent molecular studies have tended to prefer an Afrobatrachia + Microhylidae grouping [51,52,88], statistical support for this clade was generally low. Furthermore, our recent analyses recovered both the Afrobatrachia + Microhylidae and Afrobatrachia + Natatanura clades, depending on the dataset used [89]. The data used here, the longest molecular datasets so far applied to afrobatrachian phylogeny, support the Afrobatrachia + Microhylidae hypothesis. Although BP support for this node from the AA-dataset was rather low (61%), the Nuc-dataset gave a relatively high BP (76%), and the Nuc- and AA-datasets had 100 and 99% BPPs, respectively.

A time tree of anurans was reconstructed using the best tree topology and the Nuc-dataset (excluding the AC-coded partition of the mt 1st codons). The resultant ages of major anuran groups are shown in Figure 2 (nodes I–XVI). The divergence time between Afrobatrachia and Microhylidae (VI) was estimated as 100 Ma with a 95% confidence interval (CI) of 82–119 Ma, while the last common ancestor of the extant afrobatrachians existed 91 Ma (CI, 74–109 Ma). These ages were slightly younger than in previous studies, although the previously suggested ages (117–143 Ma and 102–107 Ma, respectively [38,51,89-92]) were within the 95% CI ranges. The estimated split of Afrobatrachia from other ranoids (Microhylidae) at 100 Ma corresponds to the continental separation of Africa and South Africa (e.g., [93]), the last stage of the break-up of the Gondwana supercontinent, which may explain why the distribution of afrobatrachians is limited to Africa.

### Extensively rearranged mt genomes in afrobatrachians

In this study, we sequenced the whole mt genomes of four afrobatrachians representing all afrobatrachian families as follows: *Breviceps adspersus* (Brevicipitidae; genome size = 28,757 bp), *Hemisus marmoratus* (Hemisotidae; 20,093 bp), *Hyperolius marmoratus* (Hyperoliidae; 22,595 bp), and *Trichobatrachus robustus* (Arthroleptidae; 21,418 bp). The mitogenomic organizations of these afrobatrachians and other anurans are shown in Figure 1. Almost all basal anurans (= archaeobatrachians, excluding *Leiopelma* with *nd6* and *trn*P translocations [43]) have the vertebrate-type mt gene arrangement. A slight rearrangement of this order, with translocations of three *trn*s yielding the LTPF *trn* cluster, is shared by most neobatrachians (neobatrachian-type arrangement). This gene arrangement was likely present in the common ancestor of neobatrachians [38]. Within neobatrachians, extensive gene rearrangements, with duplications and/or rearrangements of *nd5*, *trn*s, and CR, have been reported in three distinct natatanuran lineages: the families Rhacophoridae + Mantellidae (= Rhacophoroidea; Figure 2 and see [4]), a part of Ranidae [47,49], and a part of Dicroglossidae (e.g., [48]). We also discovered extensively-rearranged mt genomes in afrobatrachians.

Among the afrobatrachians analyzed, the gene order of the *Hemisus* mt genome was very similar to the neobatrachian-type arrangement, except that *trn*P in the typical LTPF *trn* cluster was translocated (PLTF in *Hemisus*) and *trn*N–O_L_ and *trn*A in the typical WAN–O_L_–CY *trn* cluster were exchanged (WN–O_L_–ACY in *Hemisus*) (Figure 1). In contrast, the other three afrobatrachians showed extensive mt gene rearrangements. Their mt genomes were characterized by many duplicated genes and CRs and by pseudogenes (remnants of duplicated genes). Mainly because of these duplicated segments, the afrobatrachian mt genomes were larger than those of other vertebrates (generally 16–17 kbp (e.g., [1])). In particular, *Breviceps* has the largest known vertebrate mt genome; the second largest being 25,972 bp, with a 9 kbp duplication including CR + *12S* and *16S rrn*s, in a parthenogenetic strain of the gecko *Heteronotia binoei*[94].

In the *Breviceps* mt genome, the region consisting of LTPF *trn*s–*12S rrn*–*trn*V*–16S rrn* was tandemly triplicated. The *trn*HS_2_ segment was translocated from its original position (between *nd4* and *nd5*) into the triplicated region (between copies 2 and 3; Figure 1). Furthermore, the *trn*WN–O_L_ segment was duplicated, and an additional CR occurred between these two copies. Many of the duplicate genes became pseudogenes (one pseudo-*12S rrn*, two pseudo-*16S rrn*s, one pseudo-*trn*F, two pseudo-*trn*P, two pseudo-*trn*Vs, and one pseudo-*trn*T) or were deleted from the genome (possibly two *trn*L_2_s and one *trn*T). However, both copies of *12S rrn* and *trn*F appear to have retained their functions, because each copy has the same or quite similar nucleotide sequences (99.3% similarity for the *12S rrn*s; 100% for the *trn*Fs).

In the *Hyperolius* mt genome, the typical LTPF *trn* cluster was rearranged to PTL_2_F*,* and relatively large noncoding regions were found between *trn*P and *trn*T (1.3 kbp) and between *trn*L_2_ and *trn*F (0.4 kbp). Furthermore, the *trn*M–*nd2* segment was duplicated, with an additional CR inserted between the two copies. One copy of each gene was converted into a pseudogene (Figure 1). Similarly, in the *Trichobatrachus* mt genome, the *trn*HS_2_–*nd5* segment was duplicated, with an additional CR–LTPF *trn*s segment occurring between the copies and conversion of duplicated genes to pseudogenes (Figure 1).

The duplicated genes and rearrangement patterns differed among the afrobatrachian taxa. Thus, these extensive mitogenomic reorganizations clearly occurred independently in at least three distinct afrobatrachian lineages (i.e., breviciptids, hyperoliids, and arthroleptids; Figure 2). However, the WN–O_L_–ACY *trn* cluster, modified from the typical neobatrachian arrangement, was shared by all four afrobatrachian families and has not been found in any other vertebrate mt genome ([28], see also the Mitozoa database [95]). This gene order can only be explained by a complex rearrangement process (at least two duplication events, or one duplication and one insertion event; Additional file 5). Thus, the data strongly suggested that this arrangement occurred in the common ancestral lineage of afrobatrachians and can be regarded as a novel molecular synapomorphy for this taxon.

### Mechanisms of gene rearrangement and concerted evolution in afrobatrachian mt genomes

#### ***Mechanism of mt gene rearrangements***

Mitochondrial genomes of bilateral animals (including vertebrates) generally contain only one set of genes, a single CR, and no introns or long intergenic spacers (e.g., [1,2]). In such genomes, unregulated gene rearrangement would destroy an essential single-copy gene. Thus, rearrangements in animal mt genomes are generally explained by the “duplication and deletion model” (e.g., [16,28]): first, a multi-gene (and CR) portion of the genome is duplicated, and then one duplicate gene copy becomes nonfunctional (a pseudogene) and is subsequently excised from the genome. The afrobatrachian mt genomes analyzed here had many duplicated genes, CRs, and pseudogenes, clearly indicating the occurrence of duplication-and-deletion type genomic rearrangements.

Duplications in animal mt genomes are hypothesized to mainly occur by replication errors, such as slipped-strand mispairing or asynchrony in the points of initiation and termination (e.g., [26,28]). Such replication errors only generate tandem duplications [26]; thus, the TDRL model can explain the tandemly-duplicated gene segments in afrobatrachian mt genomes. However, several non-tandemly duplicated genes and CRs in the afrobatrachian mt genomes (Figure 1) cannot be easily explained by this model. In particular, an additional copy of non-tandemly duplicated segments was sometimes positioned between other tandemly-copied segments. For example, in the *Breviceps* mt genome, an additional copy of the *trn*HS_2_ segments occurred between the tandemly triplicated LTPF *trn*s–*12S rrn*–*trn*V–*16S rrn* segments. Also, additional CRs occurred in the *Breviceps* and *Hyperolius* mt genomes between the tandemly-duplicated *trn*WN–O_L_ and *trn*M–*nd2* segments, respectively. Finally, in the *Trichobatrachus* mt genome, an additional CR–LTPF *trn*s segment existed between tandemly-duplicated *trn*HS_2_–*nd5* segments.

Previously, we proposed a model (the first tandem and second non-tandem duplication model) to explain non-tandem duplications in animal mt genomes [4]. In this model, a tandem duplication initially introduces redundant genes or CRs into a mt genome (one copy is non-essential and can be destroyed), then a non-tandem duplication (via several recombination related processes; see [4]) makes additional gene and CR copies somewhere in the tandemly-copied regions. The non-tandem copies located within tandemly-copied regions in the afrobatrachian genomes demonstrate the validity of this model.

#### ***Mechanism of concerted evolution***

Duplications of CRs are often observed in animal mt genomes, and in most cases the copied CRs are highly similar to one another (e.g., [4,27,96]). Likewise, the copied CRs in afrobatrachian mt genomes had very similar sequences [99.0% across 3,148 comparable bp in *Breviceps*, 99.6%/1,857 bp in *Hyperolius*, and 99.7%/1,390 bp in *Trichobatrachus*]. The strong nucleotide similarities of these multiple CRs may be maintained by sequence homogenization mechanisms, i.e., concerted evolution ([27]; also see below). In addition to the CRs, two *trn*PF–*12S rrn–trn*V–*16S rrn* segments (copies 1 and 3, Figure 1) seem to have experienced homogenization in the *Breviceps* mt genome. These non-neighboring copies of the tandemly triplicated segments have very high nucleotide similarity (98.5%/1,197 bp). In contrast, their neighboring regions were quite divergent (65%/1,148 bp between copies 1 and 2; 65%/1,134 bp between copies 2 and 3).

Two distinct concerted evolution mechanisms have been suggested: (1) homologous recombination and (2) illicit DNA replication accompanied by nascent strand slippage and a loop out of an extra-copied region [27]. Homologous recombination seems to cause the concerted evolution in afrobatrachian mt genomes (at least in copies 1 and 3 of the triplicated segments in *Breviceps*), because the illicit replication process cannot easily homogenize non-neighboring copies [4,29].

### Substitution rates and changes in selective pressure

Nucleotide substitution in neobatrachian mt genomes occurs more rapidly than in archaeobatrachians (e.g., [63]). Irisarri et al. [38] concluded that the accelerated substitution rates in protein-coding genes were caused by a relaxation of purifying selection in the ancestral lineage of neobatrachians. To check the occurrences of further changes in substitution rates and selective pressures within neobatrachians, we first compared the substitution rates of mt genes within five alignment categories (all mt genes, all mt protein-coding genes, *12S rrn*, 1*6S rrn*, and all *trn*s) of four neobatrachian lineages (non-ranoid neobatrachians and three major ranoid lineages: Afrobatrachia, Natatanura, and Microhylidae; Table 1) using the relative rate test. First, we compared the mt genes of ranoids and non-ranoids. The substitution rates of most ranoid mt genes (excluding *trn*s) were significantly faster than those of non-ranoids (Nos. 1–5 in Table 1: *P* ≤ 1 × 10^-7^ in all mt genes and all mt protein-coding genes [No. 1, 2]; *P* = 0.014 and 0.049 in *12Srrn* and *16Srrn* [No. 3, 4]; *P* = 0.518 in *trn*s [No. 5]), in congruence with a previous study [38]. Separate comparisons showed that there was no significant substitution rate heterogeneity among the microhylid and non-ranoid neobatrachian mt genes (Nos. 6–9, *P* > 0.05), with the exception of *trn*s (No. 10, *P* = 0.005), yet almost all mt genes of natatanurans and afrobatrachians had significantly faster substitution rates than those of non-ranoids (Nos. 11–20, *P* = 0.008 to *P* ≤ 1 × 10^-7^, excluding 16S *rrn* [No. 14, *P* = 0.119] and *trn*s [No. 15, *P* = 0.256] of natatanurans).

**Table 1:** Substitution rate comparisons of neobatrachian mitochondrial genes

**No**	**Compared genes**	**Outgroups**	**Compared lineage 1**	**Compared lineage 2**	**Substitution rate**		**Probability**	**Significance**
					**Lineage 1**	**Lineage 2**		
**Ranoides vs. non–ranoides**								* *P* < 0.05
1	All mt genes	Archaeobatrachians	Non–ranoid neobatrachians	All ranoides	0.3712	**0.3934**	≤ 1.00 × 10^–7^	*
2	All protein–coding genes	Archaeobatrachians	Non–ranoid neobatrachians	All ranoides	0.4190	**0.4440**	≤ 1.00× 10^–7^	*
3	*12S rrn*	Archaeobatrachians	Non–ranoid neobatrachians	All ranoides	0.2121	**0.2406**	0.0141	*
4	*16S rrn*	Archaeobatrachians	Non–ranoid neobatrachians	All ranoides	0.2076	**0.2232**	0.0485	*
5	All *trn*s	Archaeobatrachians	Non–ranoid neobatrachians	All ranoides	0.2786	**0.2848**	0.5180	
**Three major ranoid groups vs. non–ranoides (separate comparisons)**								* *P* < 0.05
6	All mt genes	Archaeobatrachians	Non–ranoid neobatrachians	Microhylids	**0.3712**	0.3656	0.2006	
7	All protein–coding genes	Archaeobatrachians	Non–ranoid neobatrachians	Microhylids	**0.4190**	0.4147	0.4263	
8	*12S rrn*	Archaeobatrachians	Non–ranoid neobatrachians	Microhylids	0.2121	**0.2188**	0.6312	
9	*16S rrn*	Archaeobatrachians	Non–ranoid neobatrachians	Microhylids	0.2076	**0.2110**	0.7253	
10	All *trn*s	Archaeobatrachians	Non–ranoid neobatrachians	Microhylids	**0.2786**	0.2451	0.0053	*
11	All mt genes	Archaeobatrachians	Non–ranoid neobatrachians	Natatanurans	0.3712	**0.4051**	≤ 1.00 × 10^–7^	*
12	All protein–coding genes	Archaeobatrachians	Non–ranoid neobatrachians	Natatanurans	0.4190	**0.4592**	≤ 1.00 × 10^–7^	*
13	*12S rrn*	Archaeobatrachians	Non–ranoid neobatrachians	Natatanurans	0.2121	**0.2469**	0.0084	*
14	*16S rrn*	Archaeobatrachians	Non–ranoid neobatrachians	Natatanurans	0.2076	**0.2214**	0.1198	
15	All *trn*s	Archaeobatrachians	Non–ranoid neobatrachians	Natatanurans	0.2786	**0.2911**	0.2562	
16	All mt genes	Archaeobatrachians	Non–ranoid neobatrachians	Afrobatrachians	0.3712	**0.3977**	≤ 1.00 × 10^–7^	*
17	All protein–coding genes	Archaeobatrachians	Non–ranoid neobatrachians	Afrobatrachians	0.4190	**0.4429**	4.00 × 10^–7^	*
18	*12S rrn*	Archaeobatrachians	Non–ranoid neobatrachians	Afrobatrachians	0.2121	**0.2499**	0.0046	*
19	*16S rrn*	Archaeobatrachians	Non–ranoid neobatrachians	Afrobatrachians	0.2076	**0.2389**	5.46 × 10^–5^	*
20	All *trn*s	Archaeobatrachians	Non–ranoid neobatrachians	Afrobatrachians	0.2786	**0.3119**	0.0032	*
**Comparisons among three major ranoid lineages**								* *P* < 0.0167
21	All mt genes	Archaeobatrachians	Afrobatrachians	Microhylids	**0.3977**	0.3656	≤ 1.00 × 10^–7^	*
22	All protein–coding genes	Archaeobatrachians	Afrobatrachians	Microhylids	**0.4429**	0.4147	≤ 1.00 × 10^–7^	*
23	*12S rrn*	Archaeobatrachians	Afrobatrachians	Microhylids	**0.2499**	0.2188	0.0147	*
24	*16S rrn*	Archaeobatrachians	Afrobatrachians	Microhylids	**0.2389**	0.2110	1.89 × 10^–3^	*
25	All *trn*s	Archaeobatrachians	Afrobatrachians	Microhylids	**0.3119**	0.2451	≤ 1.00 × 10^–7^	*
26	All mt genes	Archaeobatrachians	Natatanurans	Microhylids	**0.4051**	0.3656	≤ 1.00 × 10^–7^	*
27	All protein–coding genes	Archaeobatrachians	Natatanurans	Microhylids	**0.4592**	0.4147	≤ 1.00 × 10^–7^	*
28	*12S rrn*	Archaeobatrachians	Natatanurans	Microhylids	**0.2469**	0.2188	0.0192	
29	*16S rrn*	Archaeobatrachians	Natatanurans	Microhylids	**0.2214**	0.2110	0.2260	
30	All *trn*s	Archaeobatrachians	Natatanurans	Microhylids	**0.2911**	0.2451	3.00 × 10^–5^	*
31	All mt genes	Archaeobatrachians	Natatanurans	Afrobatrachians	**0.4051**	0.3977	0.0275	
32	All protein–coding genes	Archaeobatrachians	Natatanurans	Afrobatrachians	**0.4592**	0.4429	5.44 × 10^–5^	*
33	*12S rrn*	Archaeobatrachians	Natatanurans	Afrobatrachians	0.2469	**0.2499**	0.7990	
34	*16S rrn*	Archaeobatrachians	Natatanurans	Afrobatrachians	0.2214	**0.2389**	0.0233	
35	All *trn*s	Archaeobatrachians	Natatanurans	Afrobatrachians	0.2911	**0.3119**	0.0525	

Substitution rate is the mean weighted substitution rate of each lineage relative to the outgroups calculated by RRTree [81]. If the estimated *P*-value is less than 1 × 10^–7^, RRTree returns 1.00 × 10^–7^. These values are shown as ≤ 1.00 × 10^–7^. In each comparison, the faster rate is in bold. To correct for multiple testing, *P* < 0.05/3 (=0.0167) was used as the significance level in the comparisons among major ranoid lineages.

Among three major ranoid lineages, the substitution rates of all afrobatrachian mt genes were faster than those of microhylids (Nos. 21–25, *P* = 0.014 to *P* ≤ 1 × 10^-7^; Table 1). Similarly, the substitution rates of natatanurans tended to be faster than those of microhylids (Nos. 26–30, *P* = 1 × 10^-5^ to *P* ≤ 1 × 10^-7^ excluding *12S* and *16S rrn*s [Nos. 28 and 29, *P* = 0.019 and 0.211, respectively]. For Nos. 21–35, we used 0.0167 [= 0.005/3] as the significance level due to multiple testing; see [79] and Table 1). The substitution rate of all natatanuran protein-coding genes was significantly faster than that of afrobatrachians (No. 32), although other mt genes had no significant differences in substitution rates between these taxa (Nos. 31, 33–35). Overall, the relative substitution rates of the neobatrachian mt genes can be summarized as Natatanurans ≥ Afrobatrachians > Microhylidae ≈ non-ranoid neobatrachians.

To check whether the substitution rate differences of the mt protein-coding genes were caused by relaxed selective pressure and also to specify in which lineages the selective pressure had changed, we compared 19 branch models having distinct dN/dS ratios (ω) on designated branches of the best neobatrachian topology (Table 2). All 19 models produced significantly higher tree ln*L* values compared to the null model having a single ω for all branches (ω = 0.0543, –ln*L* = 150123.54, AIC = 300333.08). The best model, with the highest ln*L* and the lowest AIC (-ln*L* = 150076.11 and AIC = 300246.22: No. 15 in Table 2), had four distinct ω values and one background ω (0.054). According to this model, the mt protein genes are under strong purifying selection (ω > 0) in all anuran lineages. Selection was relaxed in three ancestral lineages of (1) Ranoides (ω = 0.093), (2) Afrobatrachia (ω = 0.107), and (3) Natatanura (ω = 0.090), yet the selection increased in all microhylid lineages (0.042). The pattern of selective pressure changes in different linages agreed rather well with the substitution rate trends among neobatrachians (Natatanurans ≥ Afrobatrachians > Microhylidae ≈ non-ranoid neobatrachians), suggesting that relaxed purifying selection was a cause of substitution rate acceleration in neobatrachian mt genomes.

**Table 2:** Comparison of branch models and branch specific changes in the selection coefficient (ω) among neobatrachian mitochondrial protein-coding genes

**No of models**	**Background ω**	**Models (designated branches having distinct ω) and estimated ω**								**–ln*L***	***P *of Likelihood ratio test (vs. null model)**	**AIC**
		**Ranoides**		**Natatanura**		**Afrobatrachia**		**Microhylidae**				
		Ancestral branch	All branches	Ancestor	All	Ancestor	All	Ancestor	All			
Null	0.0543	(single ω for all neobatrachian branches)								150123.5378	–	300333.08
1	0.0538	0.0962	–	–	–	–	–	–	–	150106.2460	3.09 × 10^–8^	300300.49
2	0.0518	–	0.0555	–	–	–	–	–	–	150118.9262	0.0099	300325.85
3	0.0502	–	0.0569 (excluding microhylids)	–	–	–	–	–	–	150107.2616	8.54 × 10^–8^	300302.52
4	0.0515	–	–	–	0.0555	–	0.0575	–	–	150114.3513	1.02 × 10^–4^	300318.70
5	0.0537	–	–	0.0918	–	0.1034	–	–	–	150107.1658	7.76 × 10^–8^	300304.33
6	0.0530	–	–	0.0921	0.0573	–	–	–	–	150107.0380	6.83 × 10^–8^	300304.08
7	0.0537	–	–	–	0.0548	0.1030	–	–	–	150118.9788	0.0105	300327.96
8	0.0537	0.0962	–	–	–	–	–	0.0596	–	150105.5203	1.50 × 10^–8^	300301.04
9	0.0546	0.0957	–	–	–	–	–	–	0.0426	150091.4881	1.21 × 10^–14^	300272.98
10	0.0498	–	0.0569	–	–	–	–	0.0595	–	150105.2662	1.16 × 10^–8^	300300.53
11	0.0517	–	0.0569	–	–	–	–	–	0.0425	150099.0388	2.29 × 10^–11^	300288.08
12	0.0532	0.0935	–	0.0887	–	0.0989	–	0.0572	–	150091.0091	2.50 × 10^–13^	300276.02
13	0.0522	0.0947	–	0.0891	–	–	0.0575	0.0586	–	150087.6890	9.94 × 10^–15^	300269.38
14	0.0532	0.0942	–	0.0901	–	–	0.0576	–	0.0423	150076.2887	1.46 × 10^–19^	300246.58
**15**	**0.0541**	**0.0931**	–	**0.0899**	–	**0.1068**	–	–	**0.0419**	**150076.1116**	**1.22 × 10**^**–19**^	**300246.22**
16	0.0523	0.0962	–	–	0.0554	0.1000	–	0.0582	–	150098.5309	3.59 × 10^–10^	300291.06
17	0.0537	0.0954	–	–	0.0554	0.0537	–	–	0.0422	150086.0065	1.93 × 10^–15^	300266.01
18	0.0497	0.0981	–	–	0.0554	–	0.0575	0.0598	–	150090.0150	9.53 × 10^–14^	300274.03
19	0.0517	0.0971	–	–	0.0554	–	0.0576	–	0.0426	150084.0983	3.01 × 10^–16^	300262.20

Background ω = ω of non–designated branches. All protein–coding gene data of all 22 neobatrachians was used in this comparison. The best model is shown in bold.

The tendency of highly rearranged mt genomes to have high nucleotide substitution rates has been observed in some animal taxa (e.g., mollusks [96-98]; ascidians [99]; lampshells [100]), and a positive correlation between substitution rate and genomic rearrangement has been demonstrated in arthropods [101,102]. Shao et al. [101] proposed that accelerated nucleotide changes lead to many illicit substitutions at the initiation and termination points of mt genome replication; such illicit initiation and termination points cause frequent tandem duplications, resulting in frequent gene rearrangements. In accordance with previous studies, most large mitogenomic rearrangements in anurans were observed in natatanurans and afrobatrachians belonging to the fast-substitution lineages. However, when we performed RRT between non-rearranged (*Hemisus*, *Limnonectes*, *Lithobates*, and two *Microhyla*) and rearranged ranoids, significant substitution rate differences were not observed in any of the five alignment categories compared here [*P* = 0.07 (all mt genes) to *P* = 0.8 (*12S rrn*)]. Given these results, we concluded that the fast nucleotide substitution rate increased the propensity of mitogenomic rearrangements but were not an absolute requirement.

### Tests of evolutionary hypotheses related to the duplicated and rearranged genes

The evolutionary trends of duplicated and rearranged genes in animal mt genomes have not been well researched because of the relative rarity of such genomic reorganizations and the lack of information on lineages with duplication and rearrangement events. Both rearranged and non-rearranged mt genomes were observed within ranoids with relatively recent divergences (< 104 Ma), and the lineages with duplications and rearrangements were well specified in this taxon. We consequently tested two evolutionary trends expected in duplicated and rearranged genes using the ranoid mitogenomic data.

#### ***Substitution rates of duplicated and rearranged mt genes***

During the evolution of duplicated genes, purifying selection is thought to be relaxed on one copy because of its redundant function; this relaxation should lead to an increased nucleotide substitution rate in one duplicate (e.g., [30-32]). To test this hypothesis, we compared the substitution rates of duplicated and rearranged genes, i.e., the triplicated *12S* and *16S rrn*s in the *Breviceps* lineage, the duplicated *nd2* in the *Hyperolius* lineage, the duplicated *nd5* in the *Trichobatrachus* lineage, and the rearranged *nd5* within a part of Ranidae (*Babina*), a part of Dicroglossidae (*Fejervarya* and *Hoplobatrachus*), and Rhacophoroidea (Figure 2) to those of the non-rearranged ranoid lineages (Table 3). The duplicated and rearranged genes did not always have faster nucleotide substitution rates. In particular, there was no significant substitution rate heterogeneity between the triplicated *12S* and *16S rrn*s in *Breviceps* and those of their non-duplicated counterparts in other ranoids (Nos. 1 and 2 in Table 3, *P* = 0.688 and 0.149). Also, the rearranged *nd5* in the *Babina* lineage showed no significant substitution rate difference compared with the non-rearranged ranoid lineages (No. 3, *P* = 0.102). The duplicated *nd5* in *Trichobatrachus* had a significantly slow substitution rate compared to the non-duplicated ranoid lineages (No. 4, *P* = 0.001), but no significant rate difference was found in the intra-afrobatrachian comparison No. 5, *P* = 0.658). Thus, faster substitution rates than in the non-rearranged lineages were only found in the rearranged *nd5* in Rhacophoroidea (No. 6, *P* = 5 × 10^-4^) and part of Dicroglossidae (No. 7, *P* ≤ 1 × 10^-7^) and in the duplicated *nd2* in *Hyperolius* (No. 8, *P* = 4 × 10^-7^).

**Table 3:** Substitution rates of duplicated and rearranged mitochondrial genes compared to non–rearranged genes

**No**	**Compared genes**	**Outgroups**	**Compared lineage 1**	**Compared lineage 2**	**Substitution rates**		**Probability**	**Significance**
			**Non–rearranged taxa**	**Rearranged taxa**	**Lineage 1**	**Lineage 2**		*
1	*12S rrn*	Non–ranoid neobatrachians	Non– *Breviceps* ranoids	*Breviceps*	**0.2364**	0.2306	0.6882	
2	*16S rrn*	Non–ranoid neobatrachians	Non– *Breviceps* ranoids	*Breviceps*	0.2187	**0.2352**	0.1495	
3	*nd5*	Non–ranoid neobatrachians	Ranoides without *nd5* rearrangement	Part of ranids (*Babina*)	0.4832	**0.5118**	0.1025	
4	*nd5*	Non–ranoid neobatrachians	Ranoides without *nd5* rearrangement	*Trichobatrachus*	**0.4832**	0.4303	0.0011	*
5	*nd5*	Non–ranoid neobatrachians	*Non–Trichobatrachus* afrobatrachians	*Trichobatrachus*	**0.4668**	0.4599	0.6581	
6	*nd5*	Non–ranoid neobatrachians	Ranoides without *nd5* rearrangement	Rhacophoroidea	0.4832	**0.5296**	5.40 × 10^–4^	*
7	*nd5*	Non–ranoid neobatrachians	Ranoides without *nd5* rearrangement	Part of dicroglossids (*Fejervarya* and *Hoplobatrachus*)	0.4832	**0.6403**	≤ 1.00 × 10^–7^	*
8	*nd2*	Non–ranoid neobatrachians	Non– *Hyperolius* ranoids	*Hyperolius*	0.5064	**0.6555**	4.38 × 10^–7^	*
9	All mt genes	Non–ranoid neobatrachians	Ranoides without *nd5* rearrangement	Rhacophoroidea	0.3769	**0.3874**	0.0024	*
10	All mt genes	Non–ranoid neobatrachians	Ranoides without *nd5* rearrangement	Part of dicroglossids (*Fejervarya* and *Hoplobatrachus*)	0.3769	**0.4045**	≤ 1.00 × 10^–7^	*
11	All mt genes	Non–ranoid neobatrachians	Non– *Hyperolius* ranoids	*Hyperolius*	0.3741	**0.4048**	≤ 1.00 × 10^–7^	*

Substitution rates is the mean weighted substitution rate of each lineage relative to the outgroups calculated by RRTree [81]. If the estimated *P*-value is less than 1 × 10^–7^, RRTree returns 1.00 × 10^–7^. These values are shown as ≤ 1.00 × 10^–7^. In each comparison, the faster rate is shown in bold.

Branch model analysis indicated that the assumed ω values of *nd2* and *nd5* on the fast substitution lineages were not substantially different from background values (*nd2*: background ω = 0.0285, *Hyperolius* branch ω = 0.0286, LRT *P* = 0.96; *nd5*: background ω = 0.0442, Rhacophoroidea ω = 0.0486, Dicroglossidae branch ω = 0.0450, *P* = 0.96; ω values were calculated under four assumed branches [= best model] + fast-evolving branches for each gene). These results indicate that gene duplication does not lead to relaxed purifying pressure on duplicated genes and to fast substitution rates in the mt genomes. Instead, the fast substitution rates of *nd2* in the *Hyperolius* lineage and of *nd5* in the Rhacophoroidea and Dicroglossidae lineages seem to simply reflect the substitution rates of the entire mt genomes. In these lineages, all of the mt genes had significantly faster substitution rates than in the non-rearranged lineages (Nos. 9–11), not just the duplicated and rearranged genes. At present, the causes of the higher substitution rates in these rearranged lineages is not obvious. The high A + T nucleotide content in the *Hyperolius* mt genome (64.8% across all genes, compared with an average of 57.8% in other ranoid mt genomes; *χ*^2^*P* = 2 × 10^-16^) may be a consequence of the high substitution rates in this genome, or vice versa.

#### ***Spatial nucleotide composition bias***

Clinal heterogeneity in the G + T nucleotide composition is known to occur in vertebrate mt genes (e.g., [33,34]). In particular, H-strand encoded genes near the O_L_ have high G + T content, while those positioned further from the O_L_ have low G + T content. This clinal variation based on distance from the O_L_ can be explained by strand-asymmetric replication, which is unique to animal mt genomes. In this replication system, the synthesis of a nascent H-strand starts at the H-strand replication origin in the CR (from right to left in Figure 1), and the synthesis of the nascent L-strand starts in the O_L_ (from left to right in Figure 1) when the nascent H-strand synthesis reaches the O_L_[5]. In this process, the template (old) H-strand results in single-stranded DNA during the L-strand synthesis. The single-stranded DNA is more prone to deamination mutations, leading to C → T (U) and A → hypoxanthine (pairing with C) → G substitutions [103,104]. Consequently, the H-strand encoding genes near the O_L_ (e.g., *co1*) have higher G + T contents (because of the low frequency of deamination of the template H-strand due to their shorter exposure times as single-strand DNA) than more remote ones.

In Ranoides, all rearranged *nd5* genes were more remote from the O_L_ compared with their original positions (Figure 1). Also, the duplicated genes in afrobatrachians were further removed from the O_L_ than their original copies. However, in almost all cases, the G + T contents of these rearranged/duplicated genes did not statistically differ from those of their non-rearranged counterparts. The average G + T contents of rearranged and non-rearranged *nd5* were 44.3 and 45.2%, respectively (*χ*^2^*P* = 0.24), those of *nd2* were 44.5 and 41.4% (*P* = 0.07), and those of *12S rrn* were 42.3 and 43.9% (*P* = 0.48). Only the G + T contents of *16S rrn* differed significantly between rearranged and non-rearranged taxa, but contrary to expectation, the rearranged *16S rrn* (in *Breviceps*) had low G + T content (40.7 and 43.9%; *P* = 0.04).

Broughton and Reneau [34] reported an increase in non-synonymous nucleotide changes (and ω) in proportion to distance from O_L_ in fish and mammal mt genomes, and they argued that this phenomenon was caused by long-term exposure of the single-stranded H-strand DNA during strand-asymmetric replication. However, as mentioned above, the estimated ω of the rearranged genes in the rearranged lineages did not differ from those in the non-rearranged lineages. Consequently, changes in G + T contents and non-synonymous substitution rates were not observed in the rearranged genes in anuran mt genomes. This result does not mean that strand-asymmetric replication and its accompanying deamination mutations do not occur in ranoid mt genomes. Deamination on the single H-strand is considered the cause of strand-specific nucleotide composition bias generally found in vertebrate mt genomes (C-rich L-strand, G-rich H-strand [103,104]), and this nucleotide composition heterogeneity was observed between the L- and H-strands of ranoid mt genomes (average G content of all H-strand coding genes was 28.3% on the H-strand and 13.5% on the L-strand). Rather, a relatively short time since the gene rearrangements (possibly for the lineages of *Babina, Breviceps, Hyperolius,* and *Trichobatrachus*), concerted evolution between duplicated genes, and/or strong functional constraints on these mt genes (suggested by very small ω on anuran mt genes) could have reduced the effects of replication on biased nucleotide substitutions via deamination in duplicated and rearranged mt genes.

If a specific evolutionary trend exists in the duplicated and/or rearranged genes, the data from these genes could negatively affect phylogenetic reconstruction, for instance, through long-branch attraction and/or nucleotide composition heterogeneity. This study found no unique evolutionary trends in these mt genes, however, supporting the use of duplicated and rearranged mt genes for phylogenetic inference.

## Conclusions

In this study, we discovered and described highly-rearranged mt genomes in afrobatrachian frogs. These genomes strongly supported the “first tandem and second non-tandem duplication model” for mitogenomic rearrangements and the “recombination-based model” for concerted evolution of duplicated mitogenomic regions. Our tests also suggested that the rearranged and duplicated mt genes did not evolve differently, suggesting no disadvantage to employing these genes for phylogenetic inference.

### Availability of supporting data

Detailed information on taxa and genes used in this study is given in Additional file 1. The full sequence data are available in Additional file 2. The aligned datasets (Nuc- and AA-datasets) used for phylogenetic tree reconstructions are available in Additional file 3. Sequence data and setting files for the molecular dating analysis are available in Additional file 4. The afrobatrachian O_L_ regions and possible gene rearrangement pathways leading to the afrobatrachian WN–O_L_–ACY *trn* cluster are illustrated in Additional file 5.

## Competing interest

The authors declare that they have no competing interests.

## Authors’ contributions

AK performed molecular lab work and analyzed the data. AK and MS wrote the paper. Both authors read and approved the final manuscript.

## Supplementary Material

Additional file 1 **Details on taxa and genes used in this study.** Taxa and genes used in this study are shown with gene sequence database accession numbers. Click here for file

Additional file 2 Sequence data used in this study. Click here for file

Additional file 3 Aligned (Nuc- and AA-) datasets used for phylogenetic tree reconstructions. Click here for file

Additional file 4 Sequence data and other setting files used in the dating analysis. Click here for file

Additional file 5 **Comparisons of the OL region between afrobatrachians and other neobatrachians and estimated gene rearrangement pathways.** Sequences and gene arrangements of the Light–strand replication origin and its neighborhood are shown and compared between afrobatrachians and other neobatrachians. Two distinct gene rearrangement pathways inferred from observed sequence conditions and two alternative rearrangement models are also shown. Click here for file
