# Supplementary material for: Afrobatrachian mitochondrial genomes: genome reorganization, gene rearrangement mechanisms, and evolutionary trends of duplicated and rearranged genes
Source: BMC Genomics. 2013 Sep 21;14:633. doi: 10.1186/1471-2164-14-633 (PMC3852066; doi:10.1186/1471-2164-14-633)
Supplement: Additional file 5 — Comparisons of the OL region between afrobatrachians and other neobatrachians and estimated gene rearrangement pathways. Sequences and gene arrangements of the Light–strand replication origin and its neighborhood are shown and compared between afrobatrachians and other neobatrachians. Two distinct gene rearrangement pathways inferred from observed sequence conditions and two alternative rearrangement models are also shown. [file 1471-2164-14-633-S5.pdf]

Additional file 5. Comparisons of OL region between afrobatrachians and other neobatrachians and estimated gene rearrangement pathway

A. Comparison of OL region between afrobatrachians and other neobatrachians

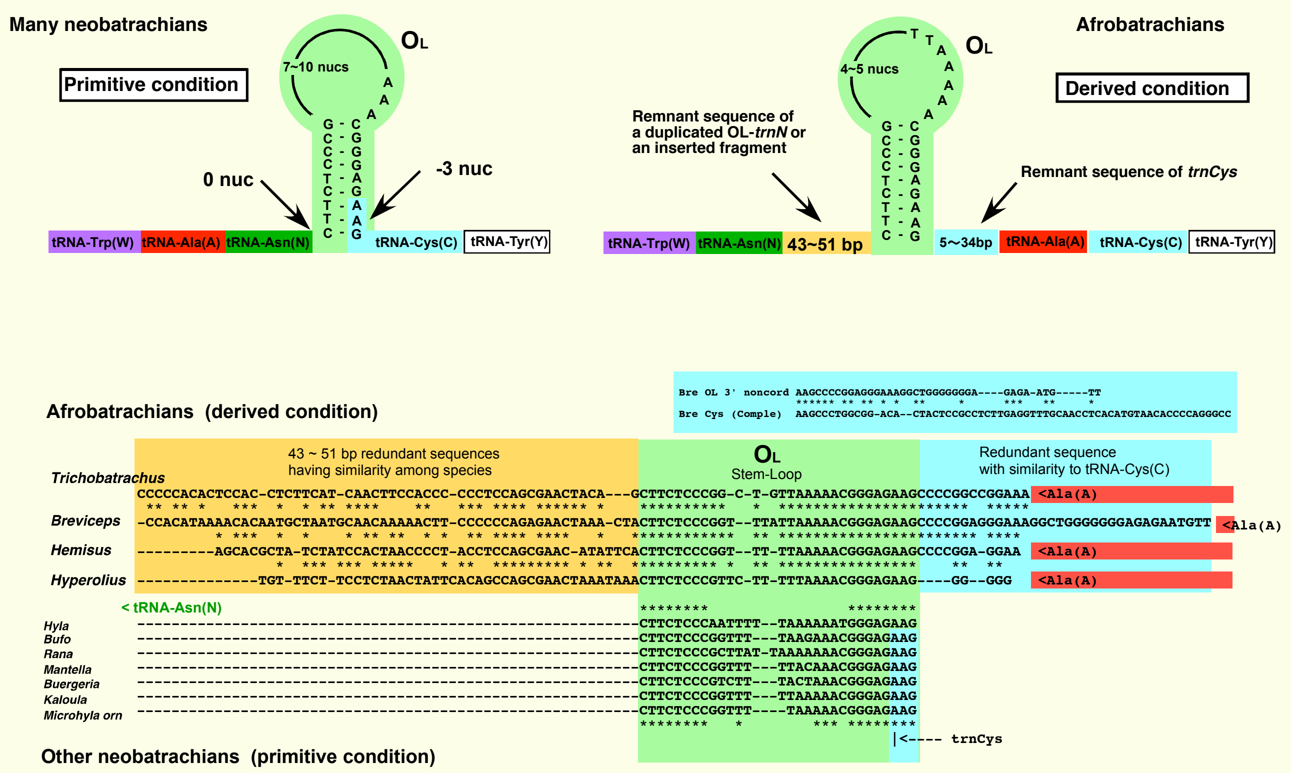

B. Two possible gene rearrangement pathways based on two alternative rearrangement models and the sequence condition of the OL region

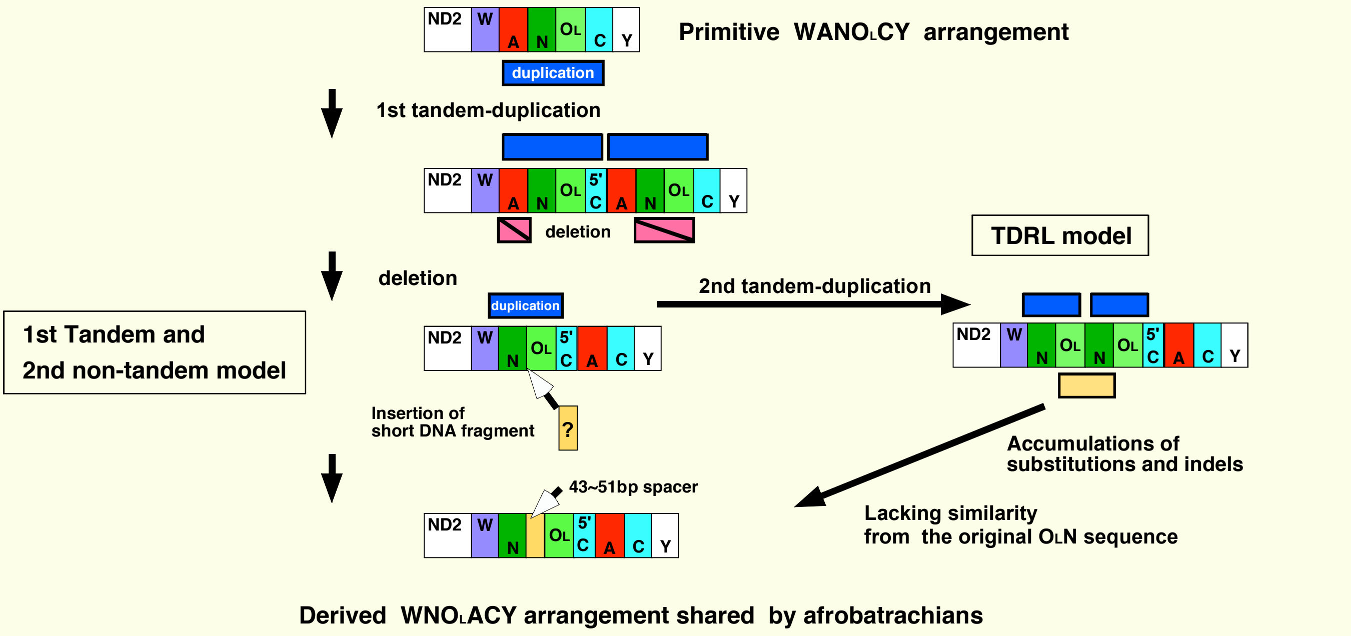

The similar 43~51 bp spacers at 5' OL observed only in afrobatrachians emphasize the common ancestry of the derived WNOLACY arrangement. The presence of the homologous spacers also suggests that the rearrangement event contains at least two distinct steps. Two alternative rearrangement pathways based on two alternative gene rearrangement models are shown.
